# Annotations

**Published:** 1888-04-14

**Authors:** 


### ANNOTATIONS.

**A Medical Prodigy at Glasgow.**

The *British Medical Journal* announces that Mr. Gulland, M.B. Edinburgh, has been appointed Examiner in Medicine and in Clinical Medicine for the ensuing year by the Glasgow University Court. *Mr. Gulland only graduated in medicine in August*, 1886. Here is a gentleman, just past the period of senior medical studentship, appointed to examine and decide upon the merits of hundreds of other men in a subject in which by no possibility can he be anything more than a tyro. Of course, Mr. Gulland may, by a proces of steady cramming, have amassed a great deal of book knowledge—knowledge which looks well on paper, and which may have gained for him the distinction of being Gold Medallist and head of his class. It is equally true that diligent attention to his duties as clinical clerk, and devotion to the more onerous claims of the house surgeon's and house physician's posts, may have given him a certain facility and readiness in the application of the rules and principles of the lecture-room; but that a man can acquire the broad wisdom and educated experience of the accomplished physician within less than two years of his graduation we emphatically deny. The examiner in medicine holds a position of serious responsibility. He should above all things be a man of sense and a man of the world. He should be able to judge men as well as written papers. Many a man can cram, and write a paper for which full marks may be gained, who is no more fit to be trusted with the lives of his fellows than with the command of a man-of-war. There are other men who cannot write papers, and whose nerves are such that their appearance before the examiners is of the sorriest kind; yet these men shall be excellent helpers by the bedside. Who is to choose for the medical profession those whose general aptitudes, as well as their academical acquirements, fit them for the duties of actual medical practice? Is this duty to be entrusted to emancipated schoolboys? Had Mr. Gulland been chosen to examine in botany or zoology there would have been less ground of complaint; but to appoint him examiner in medicine and clinical medicine is ridiculous. If the honorarium given is not sufficient to tempt men of standing from a distance there ought to be sufficient public spirit in the higher ranks of Glasgow practitioners to impel some of them to offer themselves as examiners. It was surely an unhappy day for the Glasgow University when the ripe wisdom of the late distinguished and venerated Allen Thomson was taken from it. The Scotch Universities made a wise concession to public opinion and criticism when they resolved to associate outside examiners with the ordinary professors and teachers of the students. There was thus given an adequate guarantee that the examinations would be both complete and fair. But in order that the value of such guarantee may be fully preserved, it is necessary that examiners be appointed whose experienced competence cannot be called in question.

**An Interesting Centenary at York.**

The ancient city of York has just celebrated with much sober warmth of feeling the hundredth anniversary of what is modestly called the "York Dispensary." As a matter of fact, we believe we are right in stating that the institution is much more than a dispensary. It is a "hospital," with wards for a considerable number of in-patients, and it also employs a medical staff to visit and prescribe for the sick poor at their own homes. Whatever the residents of York itself may feel towards their hospital, we have reason to know that in the towns and villages for twenty miles round the noble cathedral the "York Hospital" is regarded as a very important institution, and its physicians and surgeons are believed to have reached the very acme of medical and surgical skill and wisdom. We are glad to see that Lord Wenlock, the Dean of York, the Lord Mayor—for let it not be forgotten that the Mayor of York is a *Lord Mayor* as much as his Lordship of London—and many other influential and public-spirited inhabitants, have come forward with generous sympathy to celebrate the centenary of one of their most benevolent institutions. The hospital, or dispensary, has reached a high level of prosperity. Its invested funds amount to £17,800, as against £7,300 forty years ago. There has been a corresponding growth of need and of good work during the same period. On the motion of the Venerable Archdeacon Cresthwaite, seconded by Dr. Matterson—a familiar name at the hospital—it was resolved to make an effort to permanently increase the income by £300 per annum. To this there was an immediate and hearty response, amounting to nearly a thousand pounds. Although York is not, and has never had the ambition to be considered a "go-ahead" place, in all intellectual and benevolent work it is fully abreast of the times. The dispensary has, by long and valuable service, established a claim on all the more prosperous inhabitants of the city and surrounding districts, and we hope this interesting and appropriate celebration may inspire the institution with new life, and all its supporters with increased zeal and energy on its behalf.

**Are Midwives Despised?**

The Leamington Provident Dispensary has just held its annual meeting, and has adopted a report which, on the whole, must have been very gratifying. One item seems to have been of particular interest to the medical officers of the Dispensary, and is worthy of a passing notice by a larger public than that of Leamington. It seems that the Dispensary managers some time ago hit upon the happy idea of employing a trained midwife as a member of the regular medical staff. The experiment has been tried for a year. During that time 175 cases of confinement have been attended, of which number the medical officer has been called to 127, whilst the trained midwife has only been asked to attend 48. The report states that "the medical officers could not but express surprise and regret that the services of the midwife were not in more frequent demand. The committee had taken great care to secure the services of a highly-qualified woman for this important post, and the medical staff hoped to see the provident members availing themselves much more frequently of her skilled assistance." According to this, the poor women of Leamington prefer to be attended by a man rather than by a woman when the expense is the same. Of course there may be special reasons in this particular case; the medical officer may be better known, and therefore more popular than the midwife; or the midwife may be highly skilled, but lacking in that *suaviter in modo* which is so indispensable among the poor, as well as among the rich, at critical times. It is to be hoped there are no other reasons. There is great promise of good in the employment of trained midwives at provident dispensaries. If the medical staff were relieved of the strain of repeated night calls their energies would be saved to cope more successfully with disease during the day. Confinements being largely excluded, it might also be hoped that the most accomplished practitioners would then feel it possible to join the staff of any given provident dispensary. It cannot be said that they do so in very large numbers now. There is no doubt that in all ordinary cases of confinement a trained midwife is abundantly equal to the simple duties required of her. In a poor home she is much more helpful in many respects than a medical man. The general employment of trained midwives by provident dispensaries is strongly to be commended on many grounds, and it may be hoped that the members of those institutions will so far appreciate the efforts made by committees as to avail themselves of the skilled and sympathetic aid of midwives whenever it is possible to do so.

